# Deep-subwavelength imaging of both electric and magnetic localized optical fields by plasmonic campanile nanoantenna

**DOI:** 10.1038/srep09606

**Published:** 2015-06-05

**Authors:** Niccolò Caselli, Federico La China, Wei Bao, Francesco Riboli, Annamaria Gerardino, Lianhe Li, Edmund H. Linfield, Francesco Pagliano, Andrea Fiore, P. James Schuck, Stefano Cabrini, Alexander Weber-Bargioni, Massimo Gurioli, Francesca Intonti

**Affiliations:** 1 European Laboratory for Non-linear Spectroscopy, 50019 Sesto Fiorentino (FI), Italy; 2 Department of Physics, University of Florence, 50019 Sesto Fiorentino (FI), Italy; 3 Molecular Foundry, Lawrence Berkeley National Laboratory, Berkeley, CA 94720, USA; 4 Department of Physics, University of Trento, via Sommarive 14, 38123, Povo (TN), Italy; 5 Institute of Photonics and Nanotechnology, CNR, 00156 Roma, Italy; 6 School of Electronic and Electrical Engineering, University of Leeds, Leeds LS2 9JT, United Kingdom; 7 COBRA Research Institute, Eindhoven University of Technology, 5600 MB Eindhoven, The Netherlands

## Abstract

Tailoring the electromagnetic field at the nanoscale has led to artificial materials exhibiting fascinating optical properties unavailable in naturally occurring substances. Besides having fundamental implications for classical and quantum optics, nanoscale metamaterials provide a platform for developing disruptive novel technologies, in which a combination of both the electric and magnetic radiation field components at optical frequencies is relevant to engineer the light-matter interaction. Thus, an experimental investigation of the spatial distribution of the photonic states at the nanoscale for both field components is of crucial importance. Here we experimentally demonstrate a concomitant deep-subwavelength near-field imaging of the electric and magnetic intensities of the optical modes localized in a photonic crystal nanocavity. We take advantage of the “campanile tip”, a plasmonic near-field probe that efficiently combines broadband field enhancement with strong far-field to near-field coupling. By exploiting the electric and magnetic polarizability components of the campanile tip along with the perturbation imaging method, we are able to map in a single measurement both the electric and magnetic localized near-field distributions.

The progress made in the fabrication of nano-metamaterials at optical frequencies has led to light manipulation at the nanoscale exhibiting unprecedented artificial behaviours, such as negative or zero refractive index, optical cloaking and superluminal phenomena^1^,^2^,^3^. These nanoscale architectures, where the magnetic interaction with light can be as relevant as the electric response, are promising both for fundamental science and for cutting edge technologies, triggering the quest for novel sensing applications and field-enhancement effects at the nanoscale^4^,^5^,^6^,^7^. Therefore, a reliable imaging technique able to map both the electric and magnetic field intensities of light, which, for localized photonic modes correspond to the electric and magnetic local density of optical states (key parameters for engineering the light-matter interaction^8^,^9^,^10^) is strongly needed. Developments of different methods for measuring either the electric or the magnetic local density of states of nano-optical devices have been on going by employing scanning near-field optical microscopy (SNOM) aperture probes^11^,^12^,^13^,^14^,^15^,^16^,^17^,^18^,^19^,^20^,^21^, and more recently by using aperturless scattering SNOM or molecules which undergo detectable magnetic dipole transitions^22^,^23^,^24^.

The local SNOM probe acts as an optical antenna, converting the electromagnetic near-field into propagating radiation and vice versa, with a net energy transfer efficiency that defines the throughput of the antenna and with an imaging spatial resolution bounded by the size of the probe that interacts with the near-field. Conventional aperture probes require a trade-off between spatial resolution and throughput, since the transmission through a metal screen with a sub-wavelength hole scales as the fourth power of the hole diameter size^25^. As a matter of fact, two classes of aperture probes have been largely used: dielectric, i.e. transparent, tips with the advantage of high throughput and metallic coated tips, which show high resolution (typically ≈ 100 nm) but low throughput^26^. Although several developments have improved the SNOM sensitivity, versatility and resolution, a complete understanding of tip-light-sample interactions, and specifically which components of the electromagnetic field are collected by SNOM probes, is still lacking. In fact, this issue depends on many subtle aspects such as the exact tip geometry, the orientation of electric and magnetic dipoles with which the probes can be approximated, the light polarization, and more in general the experimental configuration.

Structures such as micro-disks, micro-waveguides, nanoparticles, nanoplasmonic resonators, and photonic crystal nanocavities (PCNs) are able to localize electromagnetic fields in very small mode volumes. Therefore, they represent successful platforms for investigating the nature of the signal detected by any SNOM probe^27^,^28^,^29^. It has been shown that, on one hand, the signal collected by dielectric tips or by metal coated circular aperture probes is directly related to the electric field intensity of the localized optical mode^15^,^18^,^30^,^31^. On the other hand, aperture metal coated tips fabricated with a wavelength dependent specific metal thickness or with a split ring can directly probe a signal that is proportional to a specific spatial component of the magnetic field intensity of light^14^,^19^,^20^. Recently, unexpected results seem to overcome this dichotomy by showing that metal coated circular aperture probes directly detect a combination of all the in-plane electric and magnetic components, with relative weights that depend both on the probe geometry and on the probe-sample distance^32^. However, this finding requires a complex modelling of the signal directly collected by the SNOM probe. The search for a method that discards the influence of the probe characteristics on the collected signal and that is capable of providing high fidelity imaging of the electromagnetic modes, has been successfully achieved by using the perturbation approach, in which the field mapping is measured by the tip induced mode spectral shift^33^,^34^. In particular, the electric or the magnetic perturbation imaging have been demonstrated by exploiting either dielectric or aperture metal coated tips^13^,^15^,^16^,^34^.

At the same time, an enormous advance in nano-imaging techniques was pursued by the introduction of concepts from plasmonics, such as surface plasmon polaritons (SPPs) confinement along metal-dielectric interfaces, which have led to the design of high-performance near-field probes^35^,^36^,^37^,^38^,^39^. With respect to metallic coated aperture probes, plasmonic tips exhibit either a better spatial resolution (at constant throughput) or larger throughput (at constant resolution). Several near-field probe geometries, integrated with plasmonic optical antennas, have been engineered and optimized with respect to size, shape and material in order to efficiently transform light from the far-field to the near-field and the other way round^40^,^41^,^42^,^43^,^44^, and for increasing the radiation efficiency of nano-optical emitters^44^. Still, they have been so far used to directly detect either the electric or the magnetic field component of light, and most of them rely on resonant structures with a limited spectral bandwidth.

In this work, we bridge together the concept of perturbation imaging method and plasmonic near field probe to achieve, in a single scan, a concomitant subwavelength imaging of the electric and magnetic near-field intensities of the optical modes localized in a PCN. We exploit the structure of a plasmonic SNOM probe that resembles the shape of a campanile bell tower (hereafter referred to as “campanile tip”) which results in a high field enhancement at the nanoscale and a simultaneous effective electric and magnetic polarizability.

## Methods

### Perturbation imaging by campanile probes

The strength of the high fidelity tip-induced perturbation imaging relies on the fact that the shift can be analytically predicted and that it is proportional to the localized mode electromagnetic field intensity^33^,^34^. Then, by mapping the spectral shift as a function of the in-plane tip position, we are able to experimentally retrieve the local density of optical states (LDOS) confined in the nanocavity. It has already been proven that a dielectric near-field probe induces a dielectric perturbation that results in a spectral red-shift of the nanocavity resonances proportionally to the localized electric field intensity^13^,^33^. In contrast, metal coated tips with a circular symmetric aperture act as diamagnetic systems inducing a mode blue-shift, which is found to be proportional to the intensity of the magnetic field component that is orthogonal to the PCN surface^15^,^16^. It is therefore tempting to combine the two spectrally opposite dielectric and diamagnetic perturbations by employing a probe featuring both an electric and a magnetic effective polarizabilities.

**Figure 1:**
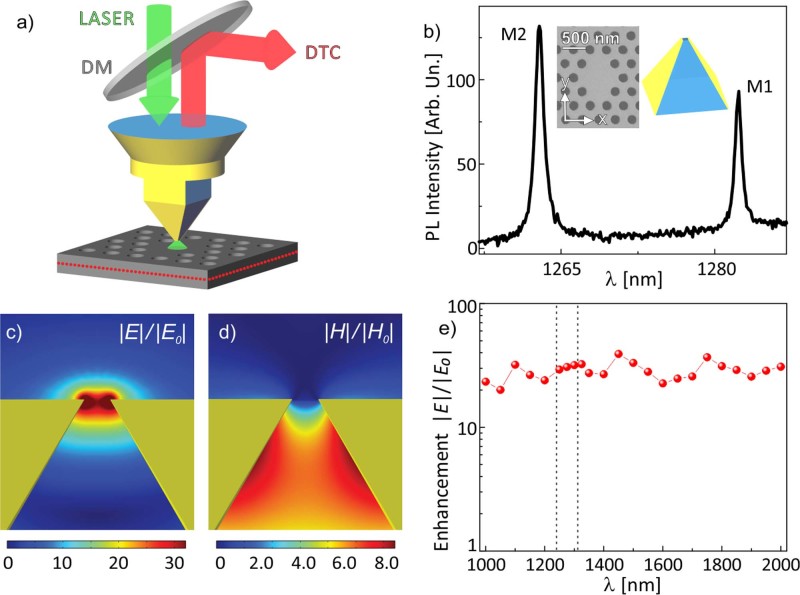
Experimental setup. (a) Schematic of the experimental setup. A room-temperature SNOM is used in illumination-collection geometry. The PL of the high-density QDs embedded in the GaAs membrane (highlighted by the red dots in the schematics) is excited by a diode laser (780 nm), coupled into the campanile near-field probe that is raster scanned at constant height on the sample surface. The PL is then collected by the same probe, reflected by a dichroic hot mirror (DM), dispersed by a spectrometer and finally detected by a cooled InGaAs array (DTC). (b) Typical PL spectrum of the investigated PCN, collected by the campanile SNOM probe and reported in the spectral range of the first two cavity modes, labelled as M1 and M2, respectively. In the insets are shown a SEM image of the PCN and a schematic of the campanile near-field probe. (c)–(d) Finite element simulation of the electric and the magnetic field distribution, respectively, evaluated at the campanile nanogap for 1.3 μm wavelength. The reported intensities correspond to the enhancement E/E_0_ and H/H_0_, respectively, where E_0_ and H_0_ are the incident electric and magnetic fields. At the nanogap E results mainly polarized along the metal separation axis, while H is polarized orthogonally to the figure plane. (e) Electric field enhancement as a function of the wavelength for a campanile tip with a 40 nm nanogap. The dashed lines highlight the experimental spectral region.

The approach of a simultaneous electric and magnetic imaging relies on the seminal idea of using concepts of discrete circuit elements, such as capacitor and inductance, to describe the tip effective interaction with the electromagnetic fields at the nanoscale^45^. The electric polarizability leads to describe dielectric probes as effective capacitors, while the diamagnetic response allows schematizing metal coated aperture tips as inductances. In this framework, the LC description of any plasmonic nanoantenna immediately leads to the possibility of addressing the electric and magnetic perturbation simultaneously. In order to prove this idea, we choose the recently developed campanile tip [see sketches in Fig. 1(a)–(b)], which is characterized by a three-dimensional tapered metal-insulator-metal (MIM) structure based on a square glass pyramid, where two opposite sidewalls are covered by gold layers (60 nm thick in this case) and terminate in a nanogap (~40 nm for our probes)^46^,^47^. The campanile tip exhibits highly efficient far-field to near-field coupling, together with an efficient light localization. Moreover, this geometry allows strong electric field enhancement confined at the nanogap, together with a magnetic field enhancement inside the tip in close proximity to the apex, as shown by the finite element calculation (evaluated at λ = 1.3 μm) reported in Fig. 1(c)–(d), respectively. In addition, the high tip throughput is effective in a broad spectral range as highlighted in Fig. 1(e). The MIM apex structure can be modeled by two metal dipole nanoantennas spaced by 40 nm dielectric material. The interaction of this system with AC electromagnetic fields can be mimicked by an equivalent LC circuit^45^. This analogy helps us to understand the simultaneous presence of both electric and magnetic polarizability. In particular, in the campanile tip the inductance is driven by a combination of electric and displacement currents flowing at the MIM apex structure. Moreover, LC circuits possess both electric and magnetic energy, which around the resonant wavelength are stored in the circuit with the same order of magnitude^48^. Therefore, the key point is that, while the induced electric dipole is parallel to the external electric field, the inductive response is diamagnetic, meaning that the associated magnetic dipole is antiparallel to the inducing magnetic field. This has strong effects on the PCN resonant wavelengths, since the electric (magnetic) dipole induced shift is expected to be toward the red (blue).

It is clear that the role of the campanile tip is twofold. On one hand, being formed by a glass core and two gold covered layers, as schematically reported in the inset of Fig. 1(b), it combines the dielectric and diamagnetic perturbations, resulting in a concomitant electric and magnetic polarizability. On the other hand, the electromagnetic field enhancement accounts for PL spectra with a good signal/noise ratio that allows monitoring with high accuracy the peak wavelength of the resonant modes. From the PL analysis, we evaluate the nanocavity localized modes spectral shift, which is related to the electric and/or magnetic field component of light depending on the effective electric and/or magnetic interaction.

### The investigated sample and the experimental setup

We consider the localized optical modes of PCNs on a 320 nm thick GaAs membrane grown by molecular beam epitaxy^49^. The photonic crystal under consideration consists of a two-dimensional triangular lattice of air holes with lattice parameter a = 301 nm and filling fraction f = 35%, and the nanocavity is formed by four missing holes organized in a diamond-like geometry, as shown in the scanning electron microscopy (SEM) image reported in the inset of Fig. 1(b) and already studied in Refs. 13, 51. In the middle of the GaAs membrane three layers of high-density (10^11^ cm^−2^) self-assembled InAs quantum dots (QDs) are grown, providing at room temperature photoluminescence (PL) in a broad spectral range centred around 1.3 μm. The PL signal can couple with the resonant photonic modes allowing us to address the optical properties of the nanocavity. The PCN is a sensitive platform to detect both dielectric and magnetic perturbations that result in a shift of the spectral resonances. The modes of the two-dimensional PCN on slab are mainly transverse electric (TE-like, that is even with respect to the z = 0 mirror plane), with the electric field polarized in the membrane plane and the magnetic field perpendicular to the plane. We consider two different TE-like modes, reported in the spectra of Fig. 1b), with a quality factor larger than 1000 (M1, the lower energy mode, and M2, the first excited mode), that exhibit almost perpendicular field spatial distributions and different polarizations^50^,^51^.

A commercial SNOM (Twinsnom by Omicron) is used in the illumination-collection geometry. The spectral response to a CW laser emitting at 1300 nm shows a FWHM of 0.11 nm. In this setup, the QDs are excited with a diode laser (780 nm) and the PL signal is coupled into the tip that is raster scanned at a constant height, smaller than 10 nm, on the sample surface. At every tip position a PL spectrum is collected, thus obtaining through the scan a hyperspectral image of the PCN. The scheme of the experimental set up is shown in Fig. 1(a).

## Results and discussion

**Figure 2:**
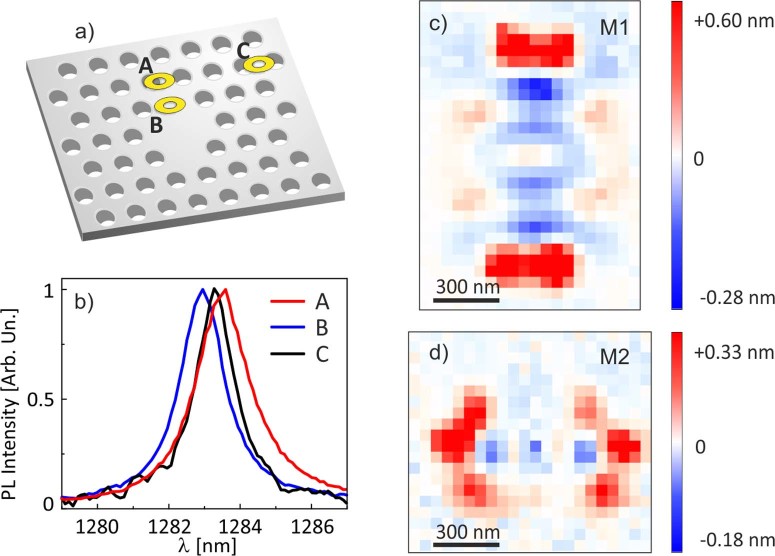
Experimental spectral shift. (a) Schematic of the investigated PCN where the three yellow spots A, B and C represent the positions where the spectra reported in (b) are collected. (b) Normalized PL signal, collected by the SNOM campanile probe for the M1 mode in the three positions reported in a). In the same scan the evidence of both a red (A) and a blue (B) tip induced spectral shift with respect to the peak wavelength detected outside the cavity (C), is clear. (c)–(d) Spectral shift distributions for the M1 and M2 mode, respectively, obtained reporting the maximum peak wavelength, evaluated by a Lorentzian fit, as a function of the tip position. The white colour, for each map, corresponds to the wavelength detected in the position of the lowest tip-induced perturbation, that is where the peak is still distinguishable from the noise. This corresponds to a position about 1 μm away from the center of the PCN.

A typical PL spectrum of the investigated nanocavity collected by the SNOM equipped with the campanile tip is reported in Fig. 1(b), referring to a position where both M1 and M2 modes are present. The peak wavelength of both modes is strongly dependent on the tip position on the sample surface. The effect is highlighted for M1 in Fig. 2(b), which reports the normalized PL spectra collected by the campanile tip at three different positions, indicated by the yellow rings labelled as A, B and C in Fig. 2(a). The PL spectrum recorded at the upper border of the PCN (position A) shows a red shift with respect to the spectrum detected outside the PCN (position C, where the intensity of the mode M1 is quite negligible). At the same time, the PL peak observed when the tip is positioned in B is blue shifted with respect to the spectrum detected in C. In fact, the tip perturbation is negligible when the near-field probe is placed in the positions where the localized electric and magnetic fields are very weak, as it happens outside the nanocavity. We perform a Lorentzian function fit of each spectrum to establish the resonant wavelength of both M1 and M2 modes as a function of tip position. Therefore, to highlight the variations that occurs during the same scan at constant height on the sample surface, we map the wavelength difference between the PL peak at every tip position and the PL peak taken outside the PCN, in a red (blue) colour scale for positive (negative) wavelength shift amplitude. The results are shown in Fig. 2(c)–(d), in which the spectral shift maps of the M1 and M2 modes are reported, respectively. Notably, a significant modulation of the spectral shift is observed in both maps. A useful characteristic of the investigated PCN in discriminating the tip induced shift of both modes is that they are more spectrally separated than the maximum observed spectral shift.

In analogy with the behaviour observed for dielectric and metal coated tips, the red (blue) wavelength shift can be interpreted as a signature of an electric (magnetic) interaction between the campanile tip and the localized photonic mode. These experimental results demonstrate the simultaneous electric and magnetic polarizability of the campanile tip as expected on the basis of the equivalence of the MIM apex structure with an LC circuit discussed in the methods section.

The campanile probe response efficiency between the external magnetic and electric fields can be evaluated as the ratio (α) between the maximum blue and red shift amplitudes. For M1 mode it is equal to |−0.28 nm| / |0.60 nm|, which gives α(M1) = (0.47 ± 0.06). For M2 mode it is |−0.18 nm| / |0.33 nm|, which gives α(M2) = (0.55 ± 0.12). These values demonstrate that the campanile tip dielectric and diamagnetic responses are of the same order of magnitude. The reported uncertainties are evaluated from the errors associated to the peak wavelength estimation provided by the Lorentzian function fit. This characteristic gives a simultaneous access to both fields with similar sensitivity, which is intriguing if one considers that the magnetic response of normally occurring materials in the optical frequency range is usually many orders of magnitude smaller than the electric response.

The different sign of the dielectric and diamagnetic response, together with the fact that for any standing wave the nodes of the electric field correspond to the maxima of the magnetic field and vice versa, are useful consequences of classical electromagnetism which allow us to clearly discriminate with high fidelity between the two field components in the same measurement. The experimental data show that the campanile probe has a concomitant positive electric and negative magnetic polarizability.

In principle, similar properties could also apply to other plasmonic nanoantennas, but the use of a campanile probe has a double benefit, as it enhances the near field coupling and also makes the magnetic and electric perturbation interaction of comparable magnitude.

**Figure 3:**
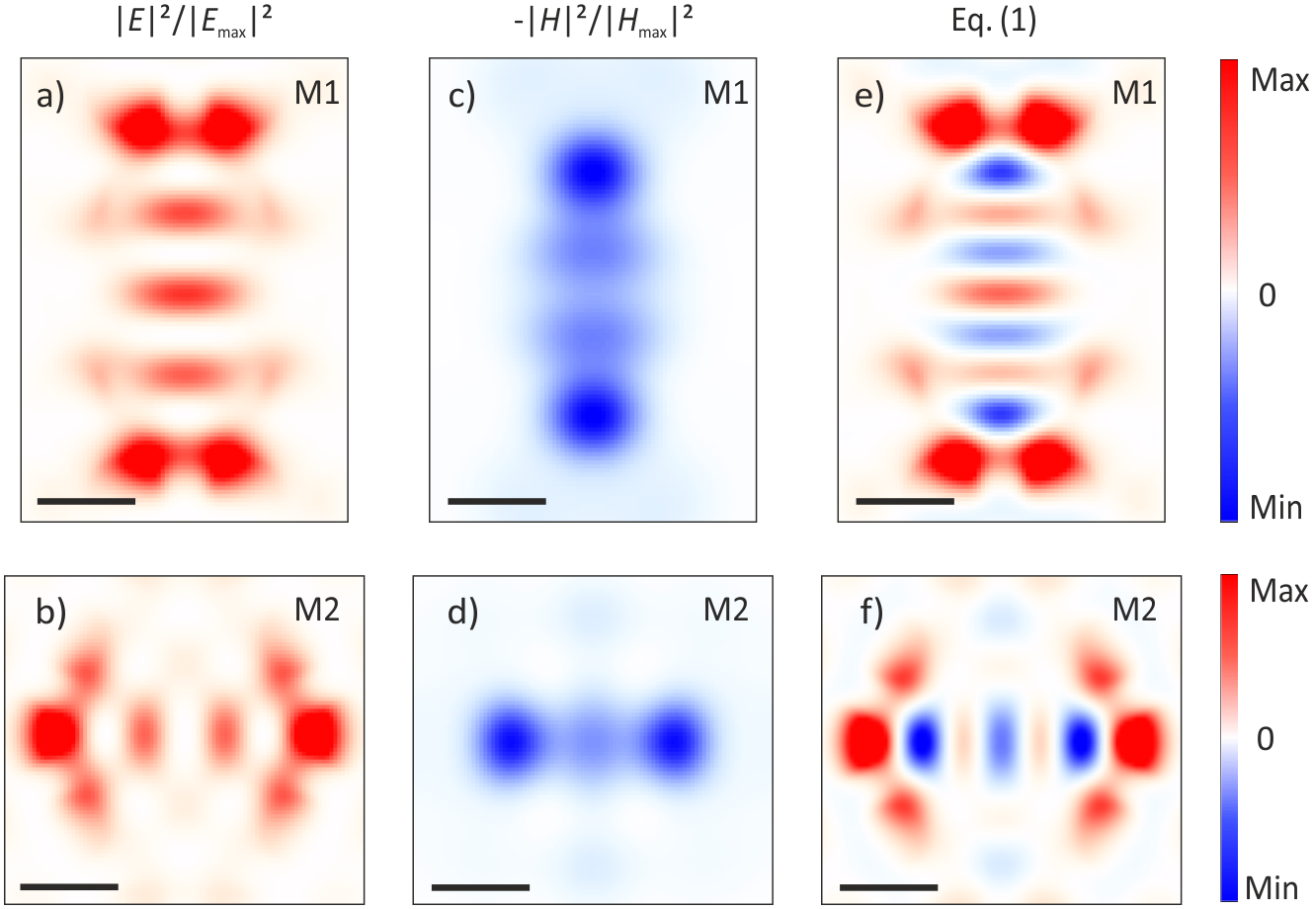
Finite Difference Time Domain calculations. (a)–(b) Electric field intensity spatial distribution normalized to the maximum for the M1 and M2 mode, respectively. (c)–(d) Intensity of the magnetic field normalized to the maximum and reported with the opposite sign for the M1 and M2 mode, respectively. (e)–(f) Distribution map evaluated using Eq.(1) with α = 0.47 and α = 0.55 for the mode M1 and M2, respectively. They nicely reproduce the experimental spectral shift maps reported in Fig. 2(c)–(d), respectively. The spatial scale bar is 300 nm for every map.

To corroborate our understanding about the imaging of both electric and magnetic contributions of the TE-like modes in a single measurement, we perform finite difference time domain (FDTD) calculations of the field distributions of the localized photonic modes. The maps associated to M1 and M2 are reported in Fig. 3(a)–(b) for the electric field intensity (red colour scale) and in Fig. 3(c) –(d) for the magnetic field intensity (blue colour scale), respectively. In order to compare the numerical calculations and the experimental data reported in Fig. 2 (c)–(d), we use the experimental ratios *α* to evaluate the expected spectral shift (Δ*λ*) distributions. These maps are constructed by a linear combination of the calculated FDTD electric and magnetic normalized intensities, assuming a negligible electric field at the position of magnetic field maxima, and conversely, that results in:
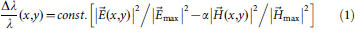
The maps calculated using α(Μ1) = 0.47 and α(Μ2) = 0.55 in Eq.(1), along with the overall constant factor chosen to match the maximum experimental spectral shift amplitude, are reported in Fig. 3 (e)–(f), respectively. Both of them closely match the experimentally measured field patterns of Fig. 2(c)–(d) in all the relevant details.

**Figure 4:**
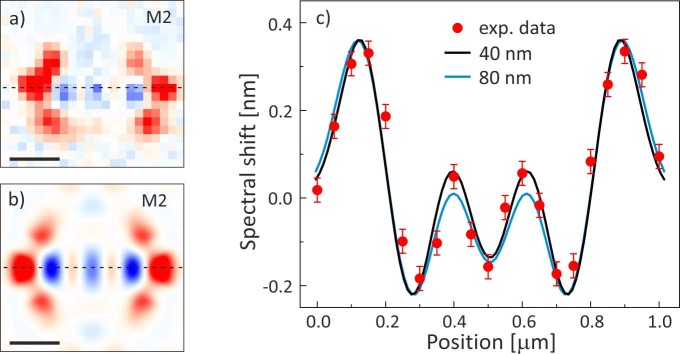
Spatial resolution of the perturbation imaging. (a)–(b) Comparison between the experimental data and the FDTD calculation using Eq.(1) for the M2 mode. The horizontal dashed lines indicate where the spatial profiles reported in (c) are evaluated. The scale bar is 300 nm. (c) Red dots represent the experimental spectral shift data collected along the dashed line in (a), compared to the theoretical profiles obtained by the convolution between the FDTD map calculated in (b) and two-dimensional Gaussian point spread functions, characterized by FWHM of 40 nm and 80 nm, and reported as black and blue line, respectively, whose intensity are normalized to the experimental spectral shift amplitude range. The error bars are provided by the Lorentzian fit output.

The experimental spatial resolution of the perturbation imaging method obtained with the campanile probe is estimated by analysing the mode map distribution along one-dimensional profile cut. We show in Fig. 4(a)–(b) the experimental spectral shift map and the FDTD calculation using Eq.(1) for the M2 mode, respectively, where the central horizontal dashed lines represent the two spatial profiles to be compared. The nanogap size at the apex of the campanile probe is expected *a priori* to result in an imaging spatial resolution of 40 nm. We check this assumption by comparing the experimental spectral shift data profile and the theoretical profiles obtained by the convolution between the FDTD map of Fig. 4(b) and two-dimensional Gaussian point spread functions, characterized by different FWHM and whose intensities are normalized to the experimental spectral shift amplitude range. The profile obtained with a Gaussian point spread function of 40 nm FWHM nicely reproduces the measured values within the experimental uncertainties, by minimizing the χ^2^ distribution. In Fig. 4(c) we report the spectral shift data profile along with the FDTD results convoluted with Gaussian functions of 40 nm and 80 nm FWHM to highlight how the former better reproduces the experimental data. Therefore, the spatial resolution achieved by the campanile probe imaging technique is about 40 nm (down to λ/30), determining a huge improvement with respect to standard dielectric or metal coated probes in photoluminescence spectral shift experiments^13^,^15^.

These data confirm that we experimentally succeed, during the same scan, in extracting high quality maps of the electric and magnetic field intensities of the localized modes with a sub-wavelength spatial resolution. There are many advantages of the perturbation method joined with campanile tip with respect to direct near-field signal collection imaging with standard coated tip employed in Ref. 32. Firstly, by perturbation imaging we are sensitive not only to the in-plane fields, thus neglecting the dominant contribution to the magnetic component of the TE-like mode, but we can access the total intensity of both the electric and the magnetic fields, which correspond to the electric and magnetic LDOS. Secondly, the mode perturbation induced by the tip polarizabilities is directly proportional to the LDOS. Therefore, we overcome the long-standing problem related to the critical interpretation of the signal collected by the SNOM probe. This not only means a high fidelity imaging but also that, after calibrating the mode shift on a well-characterized sample, it is possible to obtain a quantitative measurement of the electric and magnetic LDOS for any given nanoresonator. Finally, and importantly for many applications, the campanile tip has an enhanced throughput, which leads to the possibility of investigating also weak resonances and even individual quantum emitters. For instance in the reported measurements the collected PL signal with campanile tip is three orders of magnitude higher than the PL collected in identical experiments performed with Al-coated aperture probes with a nominal aperture of 200 nm.

The excellent agreement between the experimental data and the near-field numerical calculations clearly indicates that the campanile probe induced-perturbation imaging is able to catch all the subtle details of the electric and the magnetic intensity distributions of optical modes, with an ultra-subwavelength spatial resolution.

## Conclusions

In conclusion, we experimentally demonstrate the concomitant deep-subwavelength imaging of the electric and magnetic near-field intensities of optical modes localized in a PCN by exploiting the campanile plasmonic nano-optical probe. With respect to direct signal collection through the tip as in Ref. 32, we take advantage of both the negative-magnetic and positive-electric polarizabilities of the campanile probe for clearly splitting the electric and magnetic contributions by means of the probe induced perturbation method. The nanoscale spatial resolution is shown to be λ/30 in the telecom window wavelength range centred at 1.3 μm. In addition, our approach does not require a detailed model of the near-field probe signal collection, since the perturbation imaging high fidelity method links together the mode spectral shift and the field intensity.

Combined access to the near-field electric and magnetic components of optical modes, in conjunction with recent advances in fabricating nano-metamaterials, will help to bring together concepts from different disciplines, including nano optics, materials science and engineering, paving the way for innovative electromagnetic devices based on the interplay between the electric and magnetic optical response. One possible actual implementation could be coupling light nano-emitters (like molecules or quantum dots) to metamaterial or nanoresonator resonances for tailoring the spontaneous emission rate through electric and/or magnetic dipoles interaction, by exploiting the knowledge of electric and magnetic LDOS with an outstanding spatial resolution.
